# MicroRNA-196a promotes non-small cell lung cancer cell proliferation and invasion through targeting HOXA5

**DOI:** 10.1186/1471-2407-12-348

**Published:** 2012-08-09

**Authors:** Xiang-hua Liu, Kai-hua Lu, Ke-ming Wang, Ming Sun, Er-bao Zhang, Jin-song Yang, Dan-dan Yin, Zhi-li Liu, Jing Zhou, Zhi-jun Liu, Wei De, Zhao-xia Wang

**Affiliations:** 1 Department of Biochemistry and Molecular Biology, Nanjing Medical University, Nanjing, People’s Republic of China; 2 Department of Oncology, First Affiliated Hospital, Nanjing Medical University, Nanjing, People’s Republic of China; 3 School of Life Science, Nanjing University, Nanjing, People’s Republic of China; 4 Department of Oncology, Second Affiliated Hospital, Nanjing Medical University, Nanjing, People’s Republic of China; 5 Department of Oncology, Affiliated Nanjing Hospital, Nanjing Medical University, Nanjing, People’s Republic of China

**Keywords:** Non-small cell lung cancer, miR-196a, Proliferation, Invasion, HOXA5

## Abstract

**Background:**

MicroRNAs (miRNAs) are short, non-coding RNAs (~22 nt) that play important roles in the pathogenesis of human diseases by negatively regulating gene expression. Although miR-196a has been implicated in several other cancers, its role in non-small cell lung cancer (NSCLC) is unknown. The aim of the present study was to examine the expression pattern of miR-196a in NSCLC and its clinical significance, as well as its biological role in tumor progression.

**Methods:**

Expression of miR-196a was analyzed in 34 NSCLC tissues and five NSCLC cell lines by quantitative reverse-transcription polymerase chain reaction (qRT-PCR). The effect of DNA methylation on miR-196a expression was investigated by 5-aza-2-deoxy-cytidine treatment and bisulfite sequencing. The effect of miR-196a on proliferation was evaluated by MTT and colony formation assays, and cell migration and invasion were evaluated by transwell assays. Analysis of target protein expression was determined by western blotting. Luciferase reporter plasmids were constructed to confirm the action of miR-196a on downstream target genes, including *HOXA5*. Differences between the results were tested for significance using Student’s t-test (two-tailed).

**Results:**

miR-196a was highly expressed both in NSCLC samples and cell lines compared with their corresponding normal counterparts, and the expression of miR-196a may be affected by DNA demethylation. Higher expression of miR-196a in NSCLC tissues was associated with a higher clinical stage, and also correlated with NSCLC lymph-node metastasis. *In vitro* functional assays demonstrated that modulation of miR-196a expression affected NSCLC cell proliferation, migration and invasion. Our analysis showed that miR-196a suppressed the expression of HOXA5 both at the mRNA and protein levels, and luciferase assays confirmed that miR-196a directly bound to the 3’untranslated region of HOXA5. Knockdown of HOXA5 expression in A549 cells using RNAi was shown to promote NSCLC cell proliferation, migration and invasion. Finally, we observed an inverse correlation between HOXA5 and miR-196a expression in NSCLC tissues.

**Conclusions:**

Our findings indicate that miR-196a is significantly up-regulated in NSCLC tissues, and regulates NSCLC cell proliferation, migration and invasion, partially via the down-regulation of HOXA5. Thus, miR-196a may represent a potential therapeutic target for NSCLC intervention.

## Background

Non-small cell lung cancer (NSCLC) including adenocarcinoma and squamous cell carcinoma, is the predominant form of lung cancer, and accounts for the majority of cancer deaths worldwide [1]. Despite recent advances in clinical and experimental oncology, the prognosis of lung cancer is still unfavorable, with a 5-year overall survival rate of approximately 11% [2]. Thus, a detailed understanding of the mechanisms underlying NSCLC development and progression are essential for improving the diagnosis, prevention and treatment of this disease. Recently, accumulating evidence has shown that non-coding small RNAs may be involved in NSCLC pathogenesis, providing new insights into disease biology.

MicroRNAs (miRNAs) are 21–24 nucleotide, small, non-coding RNAs that regulate gene expression by base pairing with target mRNAs in the 3′-untranslated region (3′-UTR), leading to mRNA cleavage or translational repression [3,4]. Dysregulation of miRNAs may lead to alterations in cellular differentiation, proliferation and apoptotic processes that are important in the development of cancer [5,6]. Indeed, deregulation of miRNAs is closely associated with tumor initiation, promotion and progression via the regulation of key oncogenes or tumor suppressors [7,8]. Thus, elucidating the biological consequences of miRNA dysregulation and identifying miRNA targets are critical for a complete understanding of miRNA pathways and their underlying molecular mechanisms.

The mature miR-196a is transcribed from two *miR-196a* genes, *miR-196a-1* and *miR-196a-2*. Recently, miRNA profiling studies indicate that miR-196a is overexpressed in several tumor tissues, including NSCLC [9-12]. Furthermore, an increasing number of reports indicate that miR-196a plays important roles in development, immunity and tumor pathogenesis via the targeting of specific genes [13-17]. In this study, we demonstrate that increased miR-196a expression is a characteristic molecular change in NSCLC and investigate the effect of increased miR-196a levels on the phenotypes of NSCLC cell lines. We also show that miR-196a may function as an oncogene by directly targeting HOXA5.

## Methods

### Patient and tissue samples

Paired NSCLC and adjacent non-tumor lung tissues were obtained from 34 consecutive patients who underwent primary surgical resection of NSCLC with informed consent between 2006 and 2007 at First Affiliated Hospital of Nanjing Medical University, China. Surgically laser capture micro-dissected NSCLC and adjacent normal tissues were immediately snap-frozen in liquid nitrogen and stored at −80°C until total RNA was extracted. Tumor samples were at least 80% composed of viable-appearing tumor cells on histological assessment. The pathological stage, grade, and nodal status were appraised by an experienced pathologist. Clinicopathologic characteristics including tumor-node-metastasis (TNM) staging had been collected. The study was approved by the Research Ethics Committee of Nanjing Medical University, China.

### Cell lines and culture conditions

Four NSCLC adenocarcinomas cell lines (A549, SPC-A1, NCI-H1650, NCI-H1299) , a NSCLC squamous carcinomas cell line (SK-MES-1), a normal human bronchial epithelial cell line (16HBE), and a human embryonic kidney cell line (HEK293T) were purchased from the Institute of Biochemistry and Cell Biology of the Chinese Academy of Sciences (Shanghai, China). Cells were cultured in RPMI 1640 or DMEM (GIBCO-BRL) medium supplemented with 10% fetal bovine serum (10% FBS), 100 U/ml penicillin, and 100 mg/ml streptomycin (invitrogen) in humidified air at 37°C with 5% CO2.

### RNA extraction and qRT-PCR analyses

Total RNA was isolated with TRIzol reagent (Invitrogen, Carlsbad, CA, USA) according to the manufacturer’s protocol. qRT-PCR assays were performed to detect miR-196a and HOXA5 expression using the PrimeScript RT reagent Kit and SYBR Premix Ex Taq (TaKaRa, Dalian, China) according to the manufacturer’s instructions.

The relative level of miR-196a was determined by qRT-PCR using gene specific primers. U6 was validated as the normalizer, since which expression showed minimal variation in different cell lines and cancer specimens. The RT primers were designed as follows: miR-196a, 5’ GTCAGAAGGAATGATGCACAGCCAACAACA 3’ and U6, 5’ AACGCTTCACG AATTTGCGT 3’. The RT reaction was carried out under the following conditions: 42°C for 15 min; 85°C for 5 sec; and then held on 4°C. After the RT reaction, the complementary DNA products were diluted at 1:100, and 1ul of the diluted complementary DNA was used for subsequent qRT-PCR reactions.

The PCR primers for mature miR-196a or U6 were designed as follows: miR-196a sense, 5’ CGTCAGAAGGAATGATGCACAG 3’ and reverse, 5’ ACCTGCGTAGGTAGTTTCATGT 3’; U6 sense, 5’ CTCGCTTCGGCAGCACA 3’ and reverse, 5’ AACGCTTCACGAATTTGCGT 3’. The PCR reaction was conducted at 95°C for 30 s and followed by 40 cycles of 95°C for 5 s and 60°C for 34 s in the ABI 7500 real-time PCR system (Applied Biosystems, Foster City, CA, USA). The qRT-PCR results were analyzed and expressed as relative miRNA expression of CT (threshold cycle) value, which was then converted to fold changes.

For analysis of HOXA5 mRNA expression, 100 ng total RNA was reverse transcribed in a final volume of 10 μl using random primers under standard conditions. HOXA5-specific primers were designed as follows: sense, 5’ TCTCGTTGCCCTAATTCATCTTTT3’ and reverse, 5’ CATTCAGGACAAAGA GATGAACAGAA 3’. To verify integrity of HOXA5 expression, GAPDH gene was used as an internal control and the sequences of primers were as follows: sense, 5’ GGGAGCCAAAAGGGTCAT 3’ and reverse, 5’ GAGTCCTTCCACG ATACCAA 3’. The relative levels of HOXA5 mRNA were calculated based on the difference between amplification of HOXA5 and GAPDH mRNA using the 2^-Δ^ct method. All experiments were performed three times with three technical replicates.

### CpG island identification and methylation analysis

Using the CpG Island Searcher Web tool (http://www.ebi.ac.uk/Tools/emboss/ cpgplot/index.html), a CpG island was identified upstream (−500 bp) of the region encoding miRNA-196a-1. For determination of the methylation status of the CpG island, genomic DNA prepared from 16HBE cells, was modified by sodium bisulfite (EZ DNA Methylation Kit , Zymo Research), followed by PCR using the sense primer 5’ TTTTTTAGGATAGGAGGGGAT 3’ and reverse, 5’ TCTAAATCCTTAACCCC CTAAC 3’ , respectively. PCR-amplified product was transformed into E.coli DH5α cells. Subsequently obtained plasmids were subjected to sequencing.

### Treatment of 16HBE cells with 5-aza-2-deoxy-cytidine (5-aza-CdR)

16HBE cells (2.5 × 10^5^) were seeded into six-well culture plate on day 0 and exposed to 0, 2 or 5 μM 5-aza-CdR(Sigma-Aldrich)for day 1 to day 3. Thereafter, the cells treated with 5-aza--CdR were harvested on day 3 and used for detection of miR-196a expression or methylation analysis.

### Transfection of NCSCL cells

All plasmid vectors (pCDNA/miR-196a and pCDNA/miR-NC ) for transfection were extracted by DNA Midiprep or Midiprep kit (Qiagen, Hilden, Germany). Mature miR-196a mimics and miR-196a inhibitors (Anti-miR-196a) were purchased from Sigma-Aldrich and Applied Biosystems, AB, Ambion, respectively. The appropriate negative controls (miR-NC or anti-miR-NC) were synthesized by GenePharma, Shanghai, China. HOXA5-siRNA named si-HOXA5 and non-specific control siRNA (si-NC) were purchased from genechem, Shanghai, China. The pCDNA/miR-196a, mature miR-196a mimics or si-HOXA5 was transfected into cultured A549 cells respectively, and miR-196a inhibitors were transfected into cultured SPC-A1 cells. A549 or SPC-A1 cells were grown on six-well plate to confluence and transfected using Lipofectamine2000 (Invitrogen) according to the manufacturer’s instructions. Fourty-eight hours after transfection, cells were harvested for qRT-PCR analyses or Western blot.

### Cell proliferation assays

Cell proliferation was monitored using Cell Proliferation Reagent Kit I (MTT) (Roche Applied Science). MiR-196a inhibitors transfected SPC-A1 cells (3000/well), and pCDNA/miR-196a or si-HOXA5 transfected A549 cells (2000/well) were allowed to grow in 96-well plates. Cell proliferation was documented every 24 h following the manufacturer’s protocol. All experiments were performed in quadruplicate. For the colony formation assay, a total of 500 miR-196a inhibitors transfected SPC-A1, pCDNA/miR-196a or si-HOXA5 transfected A549 cells were placed in a fresh six-well plate and maintained in media containing 10% FBS, replacing the medium every 4 days. After 14 days, cells were fixed with methanol and stained with 0.1% crystal violet (sigma). Visible colonies were manually counted. Triplicate wells were measured in each treatment group.

### Flow-cytometric analysis of apoptosis

SPC-A1 cells transiently transfected with miR-196a inhibitors or anti-miR-NC, were harvested at 48 h after transfection by trypsinization. After the double staining with FITC-Annexin V and Propidium iodide (PI), the cells were analyzed with a flow cytometry (FACScan®; BD Biosciences) equipped with a CellQuest software (BD Biosciences) [18]. Cells were discriminated into viable cells, dead cells, early apoptotic cells, and apoptotic cells, and then the relative ratio of early apoptotic cells were compared to control from each experiment. All of the samples were assayed in triplicate.

### Hoechst staining assay

SPC-A1 cells transfected with miR-196a inhibitors were cultured in six-well plates, and were incubated with Hoechst 33342 solution (50 ng/ml, Sigma, St Louis, MO, USA) for 10 min at room temperature. Cells were then washed twice with PBS and changes in nuclear morphology were detected by fluorescence microscopy using a filter for Hoechst 33342 (365 nm). For quantification of Hoechst 33342 staining, the percentage of Hoechst -positive nuclei per optical field (at least 50 fields) was counted in three independent experiments.

### Cell migration and invasion assays

For the migration assays, 24 h after transfection, 5 × 10^4^ cells in serum-free media were placed into the upper chamber of an insert (8-μm pore size, millepore). For the invasion assays, 1 × 10^5^ cells in serum-free media were placed into the upper chamber of an insert coated with Matrigel(Sigma-Aldrich, USA). Media containing 10% FBS were added to the lower chamber. After several hours of incubation, the cells remaining on the upper membrane were removed with cotton wool, whereas the cells that had migrated or invaded through the membrane were stained with methanol and 0.1% crystal violet, imaged, and counted using an IX71 inverted microscope (Olympus, Tokyo, Japan). Experiments were independently repeated three times.

### Bioinformatics methods

The miRNA targets predicted by computer-aided algorithms were obtained from pictar (http://pictar.mdc-berlin.de/cgi-bin/new_PicTar_vertebrate.cgi.), targetscan (http://www.targetscan.org) and mirbase targets (http://microrna. sanger.ac.uk/cgi-bin/targets/v5/search.pl).

### Plasmid constructs and luciferase assay

To construct a luciferase reporter vector, HOXA5 3’-UTR fragment containing putative binding sites for miR-196a was amplified by PCR using the following primers: sense-5’-GATGTTTTAACTTATTTATATGAAG-3’ and reverse-5’-CAA ATATTGTCCAAGTCTGGCTGTT-3’. The PCR product was subcloned downstream of the luciferase gene in the pLUC Luciferase vector (Ambion, Inc.,Austin, TX, USA) and named HOXA5-3’-UTR-WT. Sitedirected mutagenesis of the miR-196a target site in the HOXA5-3’-UTR was performed using the Quick-change mutagenesis kit (Stratagene, Heidelberg, Germany) and named HOXA5 -3’-UTR-Mut, in which 3’-UTR-WT was used as a template. For the mutated construct, the miR-196a target site 5’- GATATTTTTATTCAAA CTACCTA -3’ was substituted with a 5’- GATATTTTTATTCTTTGATGGAA -3’ fragment.

Human HEK293T cells grown in a 48-well plate were co-transfected with 200 ng of either mock pCDNA/miR-NC or pCDNA/miR-196a, 10 ng of firefly luciferase reporter comprising wild type or mutant 3’UTR of HOXA5 gene, and 2 ng of pRL-TK (Promega, Madison, WI, USA) using Lipofectamie 2000 ( Invitrogen, USA). Cells were harvested 48 h after transfection for luciferase assay using a luciferase assay kit (Promega) according to the manufacturer’s protocol. Each experiment was repeated triplicates.

### Western blotting assay

Cells were lysed using mammalian protein extraction reagent RIPA (Beyotime) supplemented with protease inhibitors cocktail (Roche) and PMSF (Roche). Protein concentration was measured with the Bio-Rad protein assay kit. 50 μg protein extractions were separated by 12% SDS-polyacrylamide gel electrophoresis (SDS-PAGE), then transferred to 0.22 μm NC membranes(Sigma)and incubated with specific antibodies. Autoradiograms were quantified by densitometry (Quantity One software; Bio-Rad). GAPDH was used as control. GAPDH antibody and goat anti-HOXA5 (1:200) were purchased from sigma and Santa Cruz Biotechnology (Santa Cruz, CA, USA), respectively.

### Statistical analysis

Student’s t-test (two-tailed), One-way ANOVA and Mann–Whitney test were performed to analyze the data using SPSS 16.0 software. P values less than 0.05 were considered statistically significant.

## Results

### miR-196a expression is up-regulated in human NSCLC tissues

**Figure 1:**
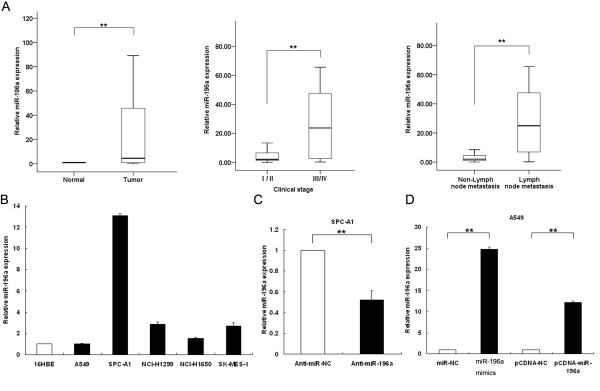
**qRT-PCR analysis of miR-196a in NSCLC tissues and matched normal tissues.** (**A**) Determining miR-196a expression in NSCLC tissues and its clinical significance. miR-196a was detected in 34 pairs of NSCLC tissues by qRT-PCR. Data are presented as fold-changes in tumor tissues relative to normal tissues. miR-196a expression was significantly higher in patients with a higher pathological stage and lymph-node metastasis. (**B**) Analysis of miR-196a expression levels in NSCLC cell lines (A549, SPC-A1, NCI-H1650, NCI-H1299 and SK-MES-1) compared with the normal bronchial epithelial cell line (16HBE) by qRT-PCR. (**C**) Analysis of miR-196a expression following treatment of SPCA1 cells with anti-miR-NC or miR-196a inhibitors by qRT-PCR. (**D**) Analysis of miR-196a expression following treatment of A549 cells with miR-NC, miR-196a mimics, pCDNA-NC or pCDNA-miR-196a by qRT-PCR. All experiments were performed in biological triplicate with three technical replicates. *P < 0.05, **P < 0.01.

The level of miR-196a was detected in 34 NSCLC samples and adjacent, histologically normal tissues by qRT-PCR, and normalized to U6. miR-196a expression was significantly up-regulated in cancerous tissues (median ratio of 22.9-fold, p < 0.01) compared with corresponding normal tissues. Furthermore, analysis of the correlation of miR-196a expression with clinical pathological features of NSCLC patients, revealed a significant association between miR-196a up-regulation and advanced pathological stage (I/II, *n* = 20) *vs* (III/IV, *n* = 14) and NSCLC lymph-node metastasis (Figure1A). We next performed qRT-PCR analysis to examine the expression of miR-196a in five human NSCLC cell lines, including both adenocarcinoma and squamous carcinoma subtypes. Of these, four cell lines (SPC-A1, NCI-H1650, NCI-H1299 and SK-MES-1) expressed higher levels of miR-196a compared with the normal, bronchial epithelial cell line, 16HBE, while A549 cells expressed relatively low endogenous levels of miR-196a (Figure1B). These results indicate that over-expression of miR-196a may play an important role in NSCLC progression and development.

### Effect of DNA methylation on miR-196a expression

**Figure 2:**
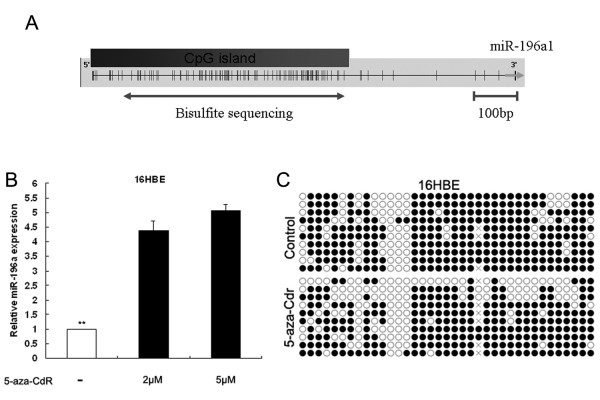
**Analysis of the correlation between methylation status and expression of miR-196a.** (**A**) Map of the CpG island position of *miR-196a-1*. Vertical ticks mark CpG sites. (**B**) The level of miR-196a expression in 16HBE cells after treatment of 5-aza-dC (0, 2, 5 μM). (**C**) The methylation status of the CpG island of *miR-196a-1* was assessed by bisulfite sequencing before and after 5-aza-dC treatment in 16HBE cells. Open and filled squares denote unmethylated and methylated CpG sites, respectively. Each row represents a single clone. *P < 0.05; **P < 0.01.

Since the expression of miR-196a was frequently up-regulated in NSCLC, we investigated whether this might be mediated by epigenetic mechanisms, including DNA demethylation. We hypothesized that epigenetic modification of CpG-rich regions within the regulatory regions of *miR-196a* may be involved in aberrant transcriptional activation. Bioinformatic analysis identified a canonical CpG island in the promoter region of the *miRNA-196a-1* loci (Figure2A); however, no canonical CpG island was found in the promoter region of the *miRNA-196a-2* loci (data not shown). Following treatment of 16HBE cells with DNA demethylating agent (5-aza-CdR), the expression of miR-196a was determined by qRT-PCR (Figure2B) and CpG island methylation was assessed by bisulfite sequencing (Figure2C). We found that miR-196a expression was significantly increased 4.4- or 5.1-fold in 5-aza-CdR treated cells compared with control, and the frequency of methylation was decreased from 78.2% to 67%. These results indicate that up-regulation of miR-196a in NSCLC cells may be affected by DNA demethylation.

### Manipulation of miR-196a levels in NSCLC cells

To selectively down-regulate or over-express miR-196a, miR-196a inhibitors or mature miR-196a mimics and their corresponding negative controls (anti-miR-NC or miR-NC, respectively) were transiently transfected into SPC-A1 or A549 cells. In addition, to stably sustain the expression of miR-196a in A549 cells, cells were transfected with pCDNA/miR-196a vector or pCDNA/miR-NC empty vector control. qRT-PCR analysis of miR-196a levels was performed 48 h post-transfection, and revealed that miR-196a expression was reduced 0.52-fold following transfection with miR-196a inhibitors and increased 24.7-fold after transfection with miR-196a mimics. Expression of miR-196a was induced 12.2-fold in stably transfected A549/miR-196a cells, compared with control (Figure1C and 1D).

### Effect of miR-196a on cell proliferation and apoptosis

**Figure 3:**
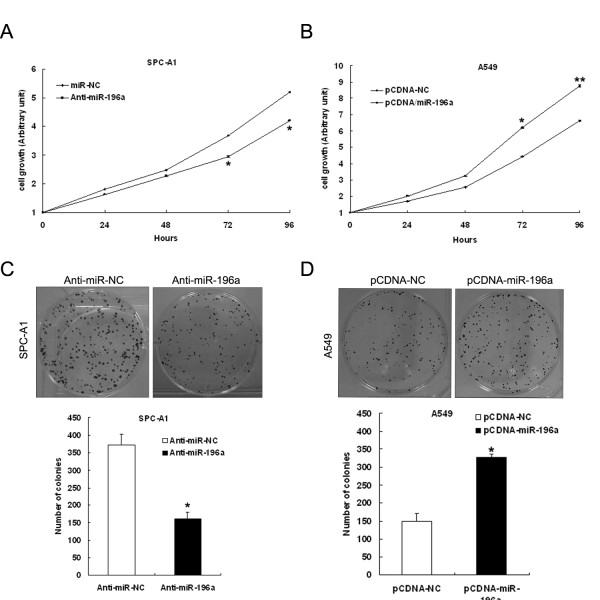
**Effect of miR-196a on cell proliferation*in vitro.*** (**A**, **B**) SPC-A1 cells were transfected with miR-196a inhibitors or anti-miR-NC, and A549 cells were transfected with pCDNA/miR-196a or pCDNA/miR-NC. MTT assay was performed to determine the proliferation of SPC-A1 or A549 cells. Data represent the mean ± S.D. from three independent experiments. (**C**, **D**) Colony-forming growth assays were performed to determine the proliferation of SPC-A1 or A549 cells. The colonies were counted and captured. *P < 0.05, **P < 0.01.

To assess the biological role of miR-196a in NSCLC, we investigated the effect of targeted knockdown or overexpression of miR-196a on cell proliferation and apoptosis. MTT assay revealed that cell growth was significantly impaired in SPC-A1 cells transfected with miR-196a inhibitors, while proliferation of A549 cells was increased in pCDNA/miR-196a transfected cells compared with controls (Anti-miR-NC or pCDNA/miR-NC, respectively) (Figure3A and 3B). Similarly, the results of colony-formation assays revealed that clonogenic survival was decreased following inhibition of miR-196a in SPC-A1 cells, and enhanced in pCDNA/miR-196a transfected A549 cells (Figure3C and 3D).

To determine whether apoptosis was a contributing factor to cell growth inhibition, we performed Hochest staining and flow-cytometric analysis of SPC-A1 cells after transfection with miR-196a inhibitors. Alteration of miR-196a expression had no significant effect on cell apoptosis compared with control cells (data not shown). Taken together, these results indicate that inhibition of miR-196a suppresses cell growth, but is not associated with induction of apoptosis.

### miR-196a promotes migration and invasion of NSCLC cells

**Figure 4:**
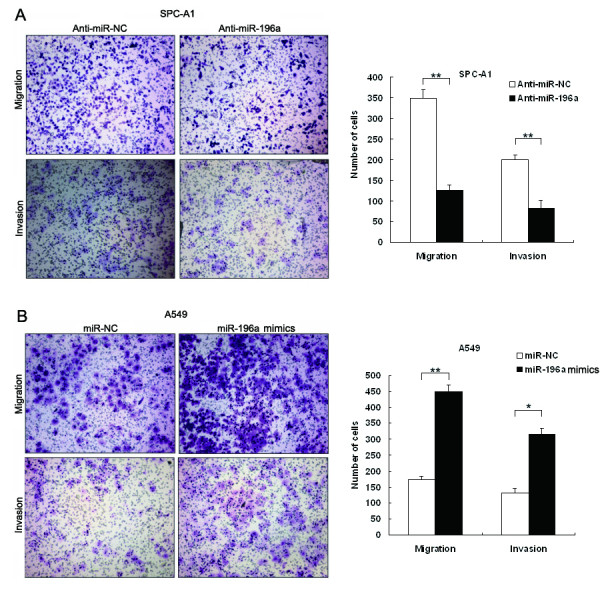
**Effect of miR-196a on cell migration and invasion*in vitro.*** (**A**, **B**) SPC-A1 cells were transfected with miR-196a inhibitors or anti-miR-NC, and A549 cells were transfected with miR-196a mimics or miR-NC. Transwell assays were performed to investigate the migratory and invasive ability of NSCLC cells. *P < 0.05 and **P < 0.01.

Cell invasion is a significant aspect of cancer progression, and involves the migration of tumor cells into contiguous tissues and the dissolution of extracellular matrix proteins. To investigate whether miR-196a had a direct functional role in facilitating NSCLC cell migration and invasion, we evaluated cancer cell invasion through Matrigel and migration through a transwell. As shown in Figure4A, inhibition of miR-196a impeded the migration of SPC-A1 cells by approximately 64% compared with control. Similarly, invasion of SPC-A1 cells was also reduced 59% following inhibition of miR-196a. Conversely, transfection of A549 cells with miR-196a mimics promoted cell migration and invasion ability ~2.5-fold (Figure4B). These data indicate that miR-196a is an onco-miRNA that can promote the migratory and invasive phenotype of NSCLC cells.

### HOXA5 is a direct target of miR-196a

**Figure 5:**
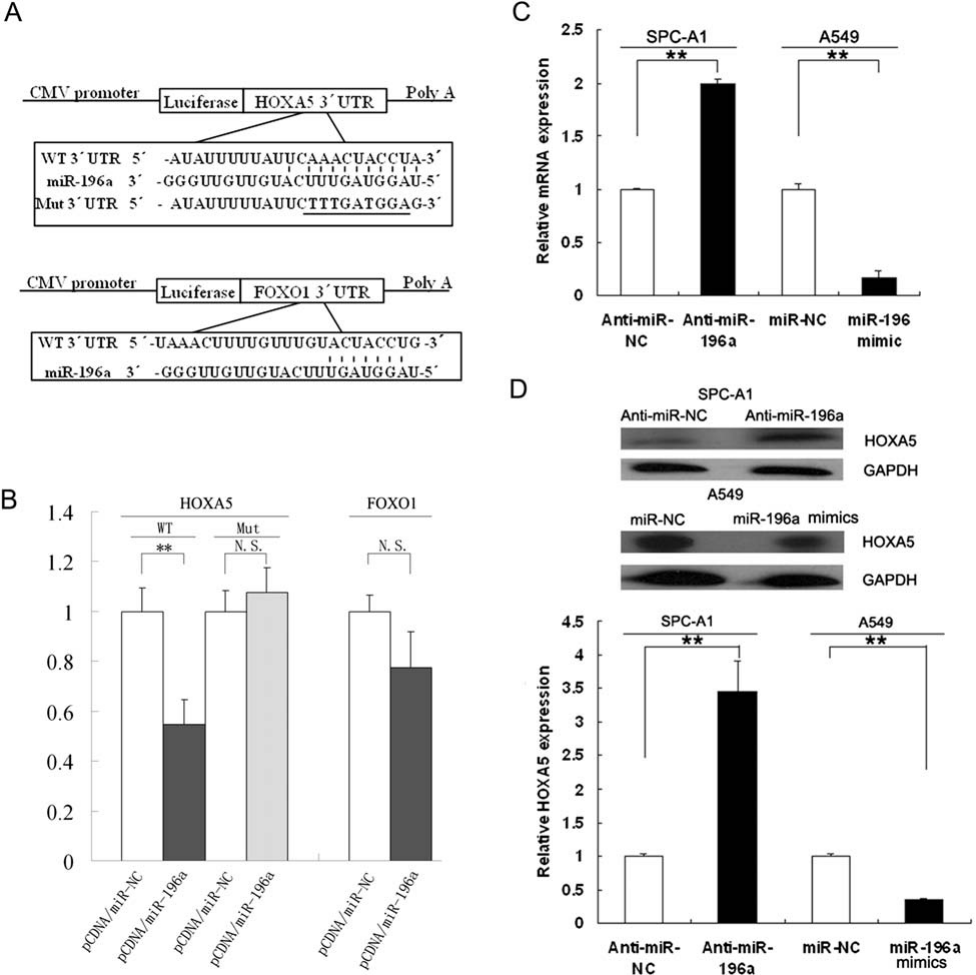
**miR-196a directly targets the*HOXA5*gene.** (**A**) A human *HOXA5* or *FOXO1* 3’ untranslated region (UTR) fragment containing the wild-type or mutant miR-196a binding sequence was cloned downstream of the luciferase reporter gene. (**B**) The luciferase reporter plasmid containing wild-type or mutant *HOXA5* and *FOXO1* 3’UTR were co-transfected into HEK-293 T cells with pCDNA-miR-196a or pCDNA-miR-NC. Luciferase activity was determined using the dual luciferase assay and shown as the relative firefly activity normalized to renilla activity. (**C**) qRT-PCR analyses of *HOXA5* mRNA level following treatment of SPCA1 cells with miR-196a inhibitors or A549 cells with miR-196a mimics. (**D**) Western blot analyses of HOXA5 protein levels following treatment of SPCA1 cells with miR-196a inhibitors or A549 cells with miR-196a mimics. GAPDH was used as control. *P < 0.05, **P < 0.01 and N.S. not significant.

To explore the molecular mechanism by which miR-196a contributes to the proliferation and invasion of NSCLC cells, we searched for potential targets as predicted by commonly cited programs such as TargetScan, PicTar and miRanda. Two candidate genes, *HOXA5* and *FOXO1*, were selected for further analysis, owing to their relatively high prediction score and their complementary structure with miRNA-196a (Figure5A). To verify whether these genes were direct targets of miR-196a, the wild-type 3’ untranslated region (UTR) of *HOXA5* and *FOXO1* were fused directly downstream of the firefly luciferase gene (pLuc). pCDNA/miR-196a was then co-transfected with various luciferase 3’ UTR constructs into HEK293T cells. MiR-196a significantly inhibited luciferase activity (~45%) of the wild-type pLuc-*HOXA5* 3’UTR reporter.

To further confirm that miR-196a-mediated reduction of luciferase activity from the pLuc-*HOXA5* 3’-UTR vector was due to direct interaction between miR-196a and its putative binding site, we mutated the miR-196a binding site by site-directed mutagenesis, resulting in pLuc-*HOXA5*-3’-UTR-Mut. As expected, suppression of luciferase activity was completely abolished in this mutant construct compared with wild-type vector (Figure5B).

We next investigated whether miR-196a could regulate HOXA5 at both mRNA and protein levels. miR-196a inhibitors or mimics were transfected into NSCLC cell lines, and the levels of HOXA5 mRNA and protein were monitored (Figure5C and 5D). qRT-PCR analysis revealed that inhibition of miR-196a in SPC-A1 cells led to increased expression of endogenous *HOXA5* mRNA compared with control. Additionally, western blot analysis showed that HOXA5 protein expression was clearly up-regulated following transfection of SPC-A1 cells with miR-196a inhibitors. In addition, enforced expression of miR-196a in A549 cells triggered a significant silencing effect on endogenous HOXA5 expression, both at the mRNA and protein level.

### Effect of HOXA5 on NSCLC cells proliferation and invasion

**Figure 6:**
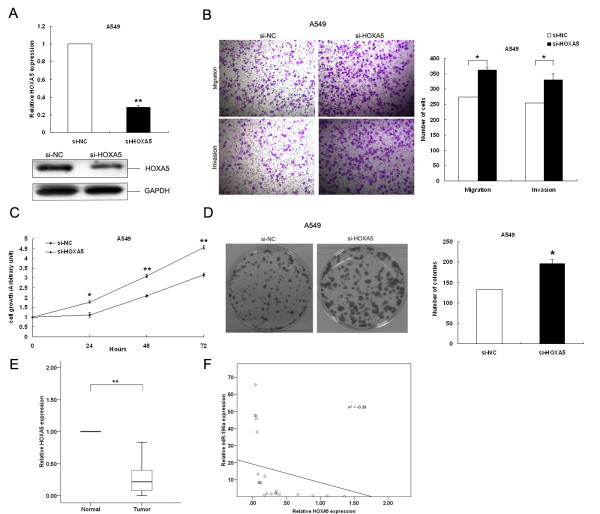
**Effect of HOXA5 on NSCLC cell proliferation, migration and invasion.** (**A**) A549 cells were transfected with si-HOXA5 or si-NC, and HOXA5 mRNA and protein levels were assessed by qRT-PCR and western blot. (**B**) Transwell assays were performed to investigate the migratory and invasive ability of NSCLC cells. (**C**, **D**) MTT assay and colony-forming growth assays were performed to determine the proliferation of si-NC or si-HOXA5 A549 cells. (**E**) The level of *HOXA5* mRNA in NSCLC tissues was analyzed by qRT-PCR. (**F**) Analysis of the relationship between miR-196a expression and *HOXA5* mRNA levels. *P < 0.05, **P < 0.01.

To determine whether HOXA5 could also inhibit NSCLC cell proliferation, migration and invasion, we performed targeted knockdown of HOXA5 expression using RNAi in A549 cells. The expression levels of HOXA5 mRNA and protein in A549/si-HOXA5 cells were significantly decreased compared with si-NC transfected cells (Figure6A). Transwell migration assays revealed that inhibition of HOXA5 promoted cell migration and invasion (Figure6B). Next, we performed MTT and colony formation assays to investigate cell growth and clonogenicity. A549 cells transfected with si-HOXA5 displayed a significantly enhanced growth ability compared with cells transfected with si-NC (Figure6C, D). These data indicate that down-regulation of HOXA5 expression promotes NSCLC cell proliferation, migration and invasion, phenocopying the over-expression of miR-196a in A549 cells.

### Downregulation of HOXA5 is inversely correlated with miR-196a expression in NSCLC

As miR-196a was over-expressed in NSCLC and targeted HOXA5 by binding to its 3’UTR, we next determined whether HOXA5 expression was negatively associated with miR-196a levels in primary NSCLC patient tissues. Analysis of *HOXA5* expression level in 34 NSCLC tissues and corresponding normal tissues by qRT-PCR revealed that *HOXA5* was significantly down-regulated in NSCLC (Figure6E). Furthermore, bivariate correlation analysis revealed that low expression of *HOXA5* was more likely correlated with high levels of miR-196a (Figure6F), suggesting that the downregulation of *HOXA5* may be due to enhanced miR-196a expression in NSCLC.

## Discussion

The aberrant expression of miR-196a is a frequent event in various cancers, suggesting that miR-196a may play an important role in tumorigenesis and tumor progression. Indeed, several key oncogenic functions have been attributed to miR-196a in the context of tumorigenesis. In esophageal cancer, miR-196a over-expression promotes cell proliferation, anchorage-independent growth and suppresses apoptosis by directly regulating ANXA1 [16]. In colorectal cancer, high levels of miR-196a were observed to activate the Akt signaling pathway, promote cancer cell detachment, migration, invasion and chemosensitivity, and increase the development of lung metastases in mice [10,17]. In previous studies from our group, we reported that aberrant over-expression of miR-196a and the resultant down-regulation of its target p27kip1, contributes to gastric carcinogenesis [11]. We also analyzed miRNA expression profiles in NSCLC patient tissues by miRNA microarray, and identified miR-196a as the most highly up-regulated miRNA compared with corresponding normal tissues [12]. Inspired by the above observation, we investigated the biological role of miR-196a and explored the molecular mechanisms by which miR-196a modulates the behavior of NSCLC cells.

In this study, we first examined the expression of miR-196a in 34 paired normal/tumor tissues from NSCLC patients, and then investigated the clinical implications. Consistent with our previous microarray analysis, miR-196a was dramatically upregulated in NSCLC tissues. Specifically, miR-196a expression was significantly higher at later stages of NSCLC development or in specimens displaying more extensive metastasis compared with their normal counterparts. This suggests that high expression of miR-196a may be involved in NSCLC carcinogenesis. In addition, we demonstrate that miR-196a expression is elevated in NSCLC cell lines compared with the 16HBE normal human bronchial epithelial cell line, with the exception of A549 cells. We also show that DNA demethylation may underlie the aberrant expression of miR-196a in NSCLC, and these findings are currently under further investigation in our laboratory.

To assess the role of miR-196a in NSCLC, we investigated the gain-or-loss of function effects of miR-196a on various aspects of NSCLC biology. First, we demonstrated that targeted knock-down of miR-196a expression in SPC-A1 cells led to significant inhibition of cell proliferation, migration and invasion. Conversely, introducing miR-196a into A549 cells, which express relatively low levels of endogenous miR-196a, induced corresponding malignant tumor cell behaviors. However, unlike previous descriptions in breast cancer, alteration of miR-196a expression did not impact apoptosis in NSCLC cells. Therefore, the functional role of miR-196a may be tissue- and cell type-specific, and the detailed mechanisms of miR-196a and its targets are worthy of further study.

Identification of putative miRNA targets is important for a complete understanding of the specific functions of miRNAs. In this study, we identify HOXA5 as a direct target of miR-196a, and demonstrate that upregulation of miR-196a significantly reduces HOXA5 expression at both transcriptional and protein level in NSCLC cells.

Mammals have four *HOX* gene clusters (*HOX A-D*), which act as master regulators that specify body patterning during embryonic development. As transcription factors, HOX proteins control a battery of target genes encoding cellular functions required for cell identity, cell growth and differentiation, as well as cell–cell and cell–matrix interactions [19-22]. Deregulation of *HOX* genes in cancer is now well established, although in general rather less is known about their function [23-28]. The homeobox protein, HOXA5, has been shown to participate in the developmental regulation of the lung. Mandeville *et al.* observed impaired postnatal lung development in *HOXA5*^*−/−*^ mice, indicating that HOXA5 has a critical role in lung ontogeny, and implying its involvement in lung maturation and function [29]. Similarly, Packer *et al.* reported that HOXA5 is likely to be involved in development and patterning of the mouse lung [30]. Moreover, dysregulation of HOXA5 expression has been associated with lung tumorigenesis and other diseases in humans [26,31]. In breast cancer, down-regulation of HOXA5 may impact *p53* gene expression, contributing to the oncogenic process [32,33]. Additionally, induction of HOXA5 is important for retinoic acid (RA)-mediated apoptosis and cellular growth inhibition acting directly downstream of RARβ, and plays an important role in RA-mediated anti-cancer activity [34,35].

Currently, the role of HOXA5 in NSCLC has not been well established. Our results reveal that inhibition of HOXA5 expression in A549 cells significantly promotes cell proliferation, migration and invasion, consistent with the results of ectopic miR-196a expression in the same cells. Furthermore, our observation of a correlation between elevated miR-196a levels and decreased HOXA5 levels in NSCLC tissues, indicates that down-regulation of HOXA5 may be a mechanism by which miR-196a exerts its oncogenic functions.

While our study provides critical insight into NSCLC pathogenesis, the existence of several limitations should be noted. First, as the number of tissue samples in this study was limited, further investigation of a larger patient cohort is essential to confirm the clinical significance of miR-196a. Second, to further understand the biological role of miR-196a in regulating NSCLC development and progression, a series of *in vivo* studies using xenograft models are required. Third, many studies have clearly indicated that one miRNA is capable of controlling multiple genes. Therefore, the observed miR-196a-mediated inhibition of cell growth and invasion is likely due to simultaneous targeting of multiple targets in NSCLC. Further studies of miR-196a will undoubtedly enhance our knowledge of how miR-196a functions in regulating NSCLC cell growth and metastasis.

## Conclusions

In summary, we demonstrate that miR-196a over-expression is a common event underlying NSCLC, and may function as an oncogene by directly targeting HOXA5. We show, possibly for the first time, that the miR-196a/HOXA5 axis regulates the proliferation and invasion of NSCLC cells. These findings may help us to better understand the pathogenesis and development of NSCLC and facilitate the development of miRNA-directed diagnostics and therapeutics against this deadly disease.

## Misc

Xiang-hua Liu, Kai-hua Lu and Ke-ming Wang contributed equally to this work and should be regarded as joint first authors.

## Competing interests

The authors declare that they have no competing interests.

## Authors’ contributions

XHL, EBZ, and MS were involved in the conception and design of the study. KHL, KMW, XHL, EBZ, MS and DDY were involved in the provision of study material and patients. JSY, ZLL, JZ and ZJL performed the data analysis and interpretation. XHL wrote the manuscript. WD approved the final version. All authors read and approved the final manuscript.

## Pre-publication history

The pre-publication history for this paper can be accessed here:

http://www.biomedcentral.com/1471-2407/12/348/prepub
